# Robotic Versus Open Placement of Hepatic Artery Infusion Pumps

**DOI:** 10.1245/s10434-025-16915-8

**Published:** 2025-01-28

**Authors:** Lauren E. Schleimer, Annie Liu, Hannah L. Kalvin, Ahmad Bashir Barekzai, Ankur P. Choubey, Joslyn Jung, Rubiya Haque, William R. Jarnagin, Vinod P. Balachandran, Ruben Geevarghese, Brett Marinelli, Mithat Gonen, Jeffrey Drebin, Peter J. Allen, Michael I. D’Angelica, Alice C. Wei, Sabino Zani, T. Peter Kingham, Michael E. Lidsky, Kevin C. Soares

**Affiliations:** 1 Hepatopancreatobiliary Service, Department of Surgery, Memorial Sloan Kettering Cancer Center, New York, NY USA; 2 Department of Surgery, Duke University Medical Center, Durham, NC USA; 3 Biostatistics Service, Department of Epidemiology and Biostatistics, Memorial Sloan Kettering Cancer Center, New York, NY USA; 4 Department of Interventional Radiology, Memorial Sloan Kettering Cancer Center, New York, NY USA

**Keywords:** Hepatic artery infusion pump, Robotic surgery, Propensity score matching weights, HAIP, Robotic HAIP placement, Liver cancer, Intrahepatic cholangiocarcinoma, Colorectal cancer liver metastasis, Liver-directed therapy

## Abstract

**Background:**

A growing number of centers offer hepatic artery infusion pump (HAIP) chemotherapy for advanced liver malignancies. While small series have demonstrated feasibility of robotic HAIP placement, comparison of outcomes with open placement is lacking. We compared outcomes after robotic versus open HAIP placement.

**Methods:**

We retrospectively reviewed HAIP placement without concurrent hepatectomy at Memorial Sloan Kettering Cancer Center from 1 January 2011 to 15 September 2022, and Duke Health from 1 November 2018 to 18 May 2023. Patients with prior liver surgery or who required catheterization of a non-standard vessel were excluded. Propensity score matching weights (PSMW) were calculated using age, sex, race, body mass index, American Society of Anesthesiologists class, neoadjuvant chemotherapy, colorectal procedure, and institution. Survey-weighted generalized linear models assessed the relationship between approach and outcomes.

**Results:**

Of 2002 consecutive HAIP placements, 819 (645 open/174 robotic) met the inclusion criteria. A higher proportion of open procedures involved combined colorectal procedures; other patient characteristics were similar. Overall, 15% of patients experienced an HAIP-specific complication and 12% required re-intervention; 2.7% had HAIP failure ≤ 90 days. After PSMW, the robotic approach had a longer operative time (*β* = 68 min, 95% confidence interval [CI] 55–81, *p* < 0.001) but shorter length of stay (*β* = − 1.8 days, 95% CI − 2.3 to 1.3, *p* < 0.001). The robotic approach was associated with increased HAIP-specific complications (odds ratio [OR] 1.72, *p* = 0.025) and re-intervention (OR 2.33, *p* < 0.001), with no difference in time to initiation of HAIP chemotherapy or HAIP failure.

**Conclusions:**

Robotic HAIP placement was associated with increased postoperative complications and significantly shorter length of stay, with similar time to initiation of HAIP therapy. There was no difference in the rate of early HAIP failure versus the open approach. These results suggest robotic HAIP placement is feasible and effective.

Hepatic artery infusion pump (HAIP) chemotherapy is an effective regional treatment modality for select patients with colorectal cancer liver metastasis and intrahepatic cholangiocarcinoma.^1–8^ Historically limited to a few select centers, the number of institutions in the United States and Europe that offer HAIP chemotherapy has been increasing rapidly.^9,10^ There is a need for contemporary data on the technical management and outcomes of HAIP placement to guide expansion of this treatment.

While HAIP placement has historically required laparotomy, the minimally invasive operative approach has gained popularity and offers several theoretical benefits, including expediting functional recovery and initiation of oncology therapy, and reducing adhesions during future liver operations.^11–14^ Although laparoscopic HAIP placement is feasible,^15,16^ technical challenges have limited its adoption.^17^ The robotics platform offers better visualization, stability, and dexterity than laparoscopy, and is ideally suited to the fine arterial dissection required for HAIP placement.

As robotics have surged in popularity across subspecialties, a rigorous safety evaluation is needed to demonstrate non-inferiority in oncologic outcomes. While this often lags years behind early adoption, it is crucial to weigh risks and benefits relative to the standard open approach.^18^ As the procedure to place an HAIP is not in itself therapeutic, the most important oncologic outcome is successful and timely initiation and continuation of HAIP therapy. Three small series have reported early experience with robotic HAIP placement,^17,19,20^ demonstrating feasibility of the approach. However, a comparison of the outcomes of robotic versus open HAIP placement has not been reported. This study reports the experience of two high-volume institutions performing robotic and open HAIP placement, and compares the outcomes of robotic versus open placement using propensity score matching weights (PSMW).

## Methods

### Data Source and Patient Population

All adult patients undergoing robotic or open HAIP placement from 1 January 2011 through 15 September 2022 at Memorial Sloan Kettering Cancer Center (MSK; New York, NY, USA) and 1 November 2018 through 18 May 2023 at Duke Health (Duke; Durham, NC, USA) were identified from prospectively maintained databases and an electronic medical records query. This retrospective study was approved by the Institutional Review Boards of MSK and Duke with a waiver of informed consent.

All HAIP implantations are performed using the standard technique as has been previously described, and included cholecystectomy if the gallbladder was present.^10,17^ Briefly, the pump pocket site is marked prior to incision and establishment of pneumoperitoneum. Open pump implantation is performed via midline laparotomy with a separate pump pocket incision, most commonly in the left mid-abdomen unless patient factors dictate placement elsewhere (e.g., left-sided ostomy). During robotic pump implantation, port sites are chosen to avoid entry into the planned pump pocket site, as this theoretically avoids additional tissue trauma within the pocket that could result in subsequent pocket-related complications. The extent of arterial dissection is similar regardless of approach, which includes circumferential arterial dissection to skeletonize the gastroduodenal artery to an extent required for safe catheter insertion (typically to the level of the pancreas) and 1–2 cm along the common and proper hepatic arteries and right hepatic artery. Any accessory or replaced hepatic arteries are routinely ligated. Lymphadenectomy is limited to the hepatic artery lymph node and any other node required to isolate the arteries as above. After arterial dissection, the pump pocket is created, the catheter is inserted transfascially, and the pump placed into the pocket with fascial sutures placed loosely through the pump anchoring rings. After the catheter is inserted, perfusion is evaluated using methylene blue for both open and robotic placements. At one of the centers (Duke), indocyanine green (ICG; 1 mg in 10 mL of sterile water) was used for selected robotic cases after extrahepatic perfusion was identified with blue dye to guide additional dissection. At this time, pneumoperitoneum is evacuated and pocket hemostasis is confirmed, and the fascial sutures are tied down. Postoperatively, nuclear medicine perfusion scans were typically obtained prior to hospital discharge.

The cohort was limited to patients who underwent HAIP placement without combined liver resection and who had no history of liver resection or liver-directed therapy (e.g., Y90 radioembolization), and included HAIP placement (open or robotic) performed in combination with a colorectal procedure, salpingo-oopherectomy, and other minor procedures. Cases involving resection of other major intra-abdominal organs (e.g., stomach, pancreas, spleen), which were only performed in combination with open HAIP placement, and those involving intraoperative radiation therapy or intraperitoneal chemotherapy were excluded. As the cannulation site has a major impact on the risk of postoperative complications,^21^ cases involving planned cannulation of a non-standard vessel (i.e., any artery other than the gastroduodenal artery) due to variant hepatic arterial anatomy or prior liver interventions were also excluded from analysis. If catheterization of a non-standard vessel was due to intraoperative complications or decision making (e.g., dissection of the gastroduodenal artery or if the gastroduodenal artery was found to be too small in caliber), such patients were included consistent with an intent-to-treat analysis.

### Outcomes

Patient and operative characteristics, as well as outcomes of interest, included operative time, estimated blood loss (EBL), length of stay (LOS), days to initiation of HAIP chemotherapy, HAIP-specific complications within 90 days, re-intervention for an HAIP-related complication within 90 days, and HAIP failure within 90 days due to technical complications.

HAIP-specific complications were categorized as arterial complications, including arterial thrombosis; arterial dissection identified during or after surgery; and bleeding or pseudoaneurysm at the cannulation site. Abnormal perfusion was defined as any extrahepatic, incomplete, or preferential perfusion requiring intervention or rendering the HAIP non-functional. Pump pocket complications included infection, hematoma, seroma, migration/flipping requiring intervention, and any other wound complication at the pump pocket site. Catheter complications included catheter occlusion and erosion. Primary device malfunctions were excluded from this analysis. Re-interventions were categorized as HAIP-related if they were performed for an HAIP-specific complication or any other complication attributable to the HAIP placement, and included diagnostic (e.g., angiographic evaluation of suspected extrahepatic perfusion or pseudoaneurysm with negative findings) and therapeutic (e.g., embolization of vessel contributing to extrahepatic perfusion or reoperation) studies.

### Statistical Analysis

PSMW was used to adjust for potential confounders,^22^ including age, sex, race, body mass index (BMI), American Society of Anesthesiologists (ASA) class, receipt of neoadjuvant chemotherapy, institution, and combined colorectal procedure. Potential confounding by combined procedures was addressed two ways. First, the PSMW comparison of operative endpoints (operative time and EBL) and LOS was limited to cases where HAIP placement was performed alone to eliminate the impact of a combined colorectal procedure on these metrics. Second, for all other endpoints, the performance of a combined colorectal procedure was included as a weighted variable in the PSMW calculation. Weighting was performed to minimize standardized mean differences (SMDs) between patients receiving open versus robotic HAIP placement while also maintaining a large sample size. Survey-weighted generalized linear models (gaussian for continuous outcomes, quasibinomial with logit link for binary outcomes) were used to assess the relationship between surgery type and outcomes of interest.

All cases with a planned robotic approach to HAIP placement that were converted to open HAIP placement intraoperatively were included in the robotic cohort, consistent with an intent-to-treat analysis. A *p*-value < 0.05 was considered statistically significant.

All analyses were conducted using R version 4.2.3 (R Foundation for Statistical Computing, Vienna, Austria).^23–25^ Results are reported in accordance with the Strengthening the Reporting of Observational Studies in Epidemiology (STROBE) best practice guidelines.^26^

## Results

**Fig. 1:**
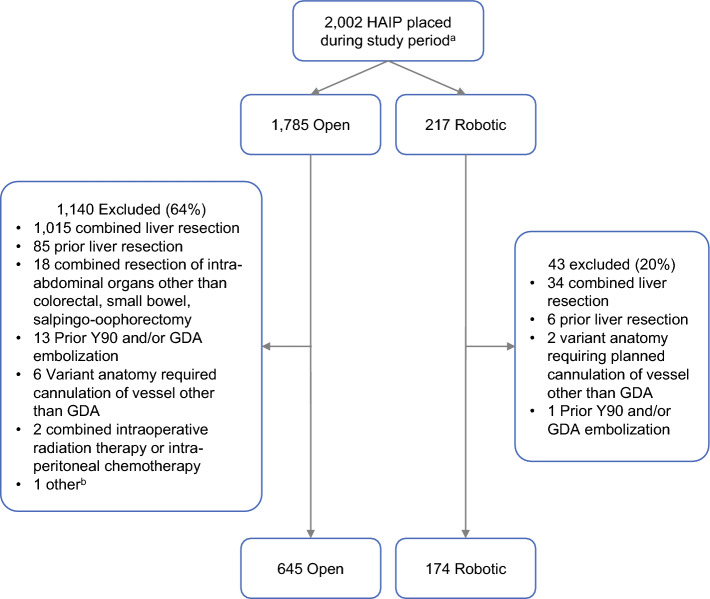
Selection of robotic and open cohorts for propensity score matching weight comparison. ^a^Study period was 1 January 2011 through 15 September 2022 at MSK; 1 November 2018 through 18 May 2023 at Duke. ^b^Unable to assess HAIP function postoperatively, as the treatment plan changed due to the results of the intraoperative biopsy; no perfusion study was performed and HAIP was never used. *HAIP* Hepatic artery infusion pump, *GDA* Gastroduodenal artery, *MSK* Memorial Sloan Kettering Cancer Center, *Duke* Duke Health

Of 2002 HAIP placements performed during the study period, 819 met the inclusion criteria for analysis, including 645 open cases and 174 robotic cases (Fig. 1); 731 were performed at MSK (601 open and 130 robotic) and 88 were performed at Duke (44 open and 44 robotic). The most common reason for exclusion in both cohorts was combined liver resection.

### Patient and Procedure Characteristics

The majority of the 819 patients in the overall cohort were male (*n* = 452, 55%) and White (*n* = 678, 87%), with a median age of 55 years (interquartile range [IQR] 47–64) at surgery (Table 1). Median BMI was 26.7 (IQR 23.5–30.8) and was similar between the two groups. Most patients (*n* = 628, 77%) underwent HAIP placement as palliative therapy for unresectable colorectal liver metastases, with a minority (*n* = 174, 21%) for intrahepatic cholangiocarcinoma and 2.1% (*n* = 17) for another pathologic diagnosis.

**Table 1:** Demographic, clinical, and operative characteristics

Characteristic	Overall [*N* = 819]	Open [*n* = 645]	Robotic [*n* = 174]	*p*-value^a^
Sex				0.7
Female	367 (45)	287 (44)	80 (46)	
Male	452 (55)	358 (56)	94 (54)	
Race				> 0.9
White	678 (87)	532 (87)	146 (87)	
Black	51 (6.6)	41 (6.7)	10 (6.0)	
Asian	33 (4.2)	26 (4.3)	7 (4.2)	
Other	16 (2.1)	12 (2.0)	4 (2.4)	
Unknown	41	34	7	
Age, years [median (IQR)]	55 (47–64)	54 (46–64)	56 (48–63)	0.5
Body mass index [median (IQR)]	26.7 (23.5–30.8)	26.7 (23.3–30.6)	26.9 (23.9–31.1)	0.5
Diagnosis				0.8
Cholangiocarcinoma	174 (21)	135 (21)	39 (22)	
Colorectal liver metastasis	628 (77)	497 (77)	131 (75)	
Other	17 (2.1)	13 (2.0)	4 (2.3)	
ASA classification				0.7
2	177 (22)	141 (22)	36 (21)	
3 or 4	642 (78)	504 (78)	138 (79)	
Neoadjuvant chemotherapy	674 (82)	523 (81)	151 (87)	0.081
Prior radiation to the liver	5 (0.6)	3 (0.5)	2 (1.1)	0.3
Any combined colorectal procedure	352 (43)	292 (45)	60 (34)	**0.011**
Combined colon resection	315 (38)	267 (41)	48 (28)	**< 0.001**
Combined rectal resection	108 (13)	94 (15)	14 (8.0)	**0.024**
Combined other operation	49 (6.0)	43 (6.7)	6 (3.4)	0.11
Appendectomy	1 (2.0)	1 (2.3)	0 (0)	
D&C	1 (2.0)	1 (2.3)	0 (0)	
Hernia repair	17 (35)	15 (35)	2 (33)	
Hysteroscopy, D&C	1 (2.0)	1 (2.3)	0 (0)	
Ileostomy reversal	2 (4.1)	2 (4.7)	0 (0)	
Salpingo-oopherectomy	23 (47)	19 (44)	4 (67)	
Small bowel resection for enterotomy	2 (4.1)	2 (4.7)	0 (0)	
Transrectal excision	2 (4.1)	2 (4.7)	0 (0)	
Conversion to open	NA	NA	9 (5.2)	
Hepatic arterial anatomy				
Standard	494 (60)	387 (60)	107 (61)	
Accessory left hepatic artery	99 (12)	76 (12)	23 (13)	
Replaced right hepatic artery	65 (7.9)	51 (7.9)	14 (8.0)	
Replaced left hepatic artery	25 (3.1)	23 (3.6)	2 (1.1)	
Accessory right hepatic artery	19 (2.3)	14 (2.2)	5 (2.9)	
Variant branching of the common hepatic artery^b^	53 (6.5)	39 (6.0)	14 (8.0)	
Replaced common hepatic artery to the superior mesenteric artery	13 (1.6)	13 (2.0)	0 (0)	
Bilateral variant vessels	37 (4.5)	29 (4.5)	8 (4.6)	
Retrograde flow through the gastroduodenal artery	2 (0.2)	2 (0.3)	0 (0)	
Other variant	12 (1.5)	11 (1.7)	1 (0.6)	
Artery cannulated				0.7
Gastroduodenal artery	805 (98)	633 (98)	172 (99)	
Other	14 (1.7)	12 (1.9)	2 (1.1)	

Bold values indicate statistically significant findings (*p* < 0.05)

Data are expressed as *n* (%) unless otherwise specified

*IQR* Interquartile range, *ASA* American Society of Anesthesiologists, *D&C* Dilatation and curettage, *NA* Not applicable

^a^Pearson’s Chi-square test, Fisher’s exact test, Wilcoxon rank-sum test

^b^Branching of the common hepatic artery was categorized as variant based on the relationship between the gastroduodenal artery, left hepatic artery, and right hepatic artery, e.g., trifurcation or early branching of left/right prior to the gastroduodenal artery

Characteristics of the robotic and open cohorts were similar (Table 1), except that a significantly higher proportion of open cases involved a combined colorectal procedure (45% open vs. 34% robotic, *p* = 0.011). Rates of neoadjuvant chemotherapy were similar for both groups (81% open vs. 87% robotic, *p* = 0.081).

Overall, most patients (*n* = 494/819, 60%) had standard hepatic arterial anatomy, with similar proportions in the robotic (60%) and open (61%) cohorts. The most common single anatomic variants were accessory left hepatic artery (*n* = 99, 12%) and replaced right hepatic artery (*n* = 65, 7.9%). The gastroduodenal artery was successfully cannulated in 98% (*n* = 805/819) of cases, as had been planned based on preoperative imaging of the arterial anatomy. Of the 174 robotic cases, 5.2% (*n* = 9) were converted to open procedures.

### Postoperative Outcomes

Overall, 15% (*n* = 120/819) of patients experienced an HAIP-specific postoperative complication, 12% (*n* = 102) required an additional procedure for an HAIP-related complication, and 2.7% (*n* = 22) had HAIP failure within 90 days of surgery (Table 2). The most common early postoperative complications were pump pocket complications (*n* = 62, 7.6%), including infection (*n* = 28, 3.4%), hematoma (*n* = 22, 2.7%), and seroma (*n* = 9, 1.1%), as well as abnormal perfusion requiring re-intervention or leading to HAIP failure (*n* = 43, 5.3%), most commonly due to extrahepatic perfusion (Table 3). Arterial complications in the first 90 days were rare (*n* = 18, 2.2%), with 1.3% (*n* = 11) of patients experiencing arterial dissection and 1.1% (*n* = 9) having bleeding or pseudoaneurysm at the cannulation site.

**Table 2:** Comparison of operative and 90-day postoperative outcomes by approach

Outcome	Overall [*N* = 819]	Unadjusted values		PSMW-adjusted values^a^		PSMW estimate^b^ robotic vs. open	95% CI	*p*-value
		Open [*n* = 645]	Robotic [*n* = 174]	Open [pN = 162]	Robotic [pN = 161]			
Operative time, min [median (IQR)]^c^	165 (138–211)	153 (131–182)	225 (197–256)	155 (131–182)	225 (197–255)	+ 68 min	+ 55 to + 81	< 0.001
Estimated blood loss, mL [median (IQR)]^c^	50 (30–125)	50 (50–150)	50 (25–100)	50 (50–126)	50 (25–100)	− 17 mL	− 44 to +11	0.2
Length of stay, days [median (IQR)]^c^	4.00 (3.00–6.00)	5.00 (4.00–6.00)	3.00 (2.00–4.00)	5.00 (4.00–6.00)	3.00 (2.00–4.00)	− 1.8 days	− 2.3 to − 1.3	< 0.001
Any HAIP-specific complication	120 (15)	87 (13)	33 (19)	21 (13)	32 (20)	OR 1.72	1.07–2.75	0.025
Abnormal perfusion	43 (5.3)	30 (4.7)	13 (7.5)	7 (4.1)	13 (8.0)	OR 2.03	0.99–4.16	0.054
Pump pocket complication	62 (7.6)	46 (7.1)	16 (9.2)	12 (7.2)	15 (9.5)	OR 1.36	0.71–2.58	0.4
Additional HAIP-related procedure	102 (12)	70 (11)	32 (18)	15 (9.4)	32 (20)	OR 2.33	1.43–3.80	< 0.001
Days to initiation of HAIP chemotherapy [median (IQR)]	17 (14–23)	17 (14–22)	15 (13–25)	17 (14–22)	15 (13–24)	− 1.4 days	− 4.4 to 1.6	0.4
90-day HAIP failure	22 (2.7)	17 (2.6)	5 (2.9)	4 (2.4)	5 (3.1)	OR 1.30	0.44–3.82	0.6

Data are expressed as *n* (%) unless otherwise specified

*IQR* Interquartile range, *HAIP* Hepatic artery infusion pump, *PSMW* Propensity score matching weights, *pN* Pseudo-N, *CI* Confidence interval, *OR* Odds ratio

^a^PSMW-adjusted values are rounded to the nearest whole number for ease of interpretation, and percentages are calculated prior to rounding; as a result, some PSMW-adjusted values may not match the percentage reported due to rounding differences

^b^Beta from a linear regression model of continuous outcomes; OR from a logistic regression model of binary outcomes

^c^Sample limited to patients who underwent HAIP placement alone without a concurrent colorectal or other procedure (*n* = 445 overall; 333 open, 112 robotic)

**Table 3:** Unadjusted HAIP-specific complications within 90 days of surgery

Category of complication	Type of complication^a^	Overall [*N* = 819]	Open [*n* = 645]	Robotic [*n* = 174]
Abnormal perfusion	Any abnormal perfusion	43 (5.3)	30 (4.7)	13 (7.5)
	Extrahepatic perfusion	38 (4.6)	26 (4.0)	12 (6.9)
	Preferential perfusion	1 (0.1)	1 (0.2)	0 (0)
	Incomplete perfusion	4 (0.5)	3 (0.5)	1 (0.6)
Arterial complication	Any arterial complication	18 (2.2)	13 (2.0)	5 (2.9)
	Bleeding or pseudoaneurysm at cannulation site	9 (1.1)	7 (1.1)	2 (1.1)
	Arterial dissection	11 (1.3)	7 (1.1)	4 (2.3)
Pump pocket complication	Any pump pocket complication	62 (7.6)	46 (7.1)	16 (9.2)
	Seroma	9 (1.1)	6 (0.9)	3 (1.7)
	Pump migration/flipping	5 (0.6)	4 (0.6)	1 (0.6)
	Infection	28 (3.4)	24 (3.7)	4 (2.3)
	Hematoma	22 (2.7)	13 (2.0)	9 (5.2)
	Extravasation	1 (0.1)	1 (0.2)	0 (0)
	Dehiscence	2 (0.2)	2 (0.3)	0 (0)
Catheter complication	Any catheter complication	2 (0.2)	2 (0.3)	0 (0)
	Catheter occlusion	1 (0.1)	1 (0.2)	0 (0)
	Catheter thrombosis	1 (0.1)	1 (0.2)	0 (0)

Data are expressed as *n* (%)

*HAIP* Hepatic artery infusion pump

^a^Patients may have experienced multiple complications in the same category

Of the 102 patients who required re-intervention, 55% (*n* = 56) were performed by interventional radiology (IR), 38% (*n* = 39) were managed with reoperation, and 6.9% (*n* = 7) received both IR and reoperation. The most common reasons for re-intervention for an HAIP-related complication were abnormal perfusion study (*n* = 44, 43%) typically managed by IR intervention, and pump pocket complications (*n* = 37, 36%) (Table 4) typically requiring operative intervention.

**Table 4:** Reason for re-intervention for HAIP-related complication within 90 days of surgery

Indication for re-intervention	*N* = 102 (%)^a^
Abnormal perfusion study^b^	44 (43)
Pump pocket complication	37 (36)
Arterial complication^b^	18 (18)
Catheter complication	1 (1.0)
Other HAIP-related intra-abdominal complication	5 (4.9)
Elective pump removal	1 (1.0)

*HAIP* Hepatic artery infusion pump

^a^Some patients had re-intervention for multiple types of complications

^b^Includes diagnostic studies with negative findings

The 30-day postoperative mortality rate was 0.5% (*n* = 4/819), with one death due to myocardial infarction on postoperative day 11, one death due to unknown causes, and two deaths related to rapid progression of disease. The 90-day postoperative mortality rate was 4.2% (*n* = 34/818; one patient had unknown vital status at 90 days). Most of the 90-day deaths were related to progression of disease, with no deaths documented as related to an HAIP-specific complication.

### Robotic Versus Open Approach

**Fig. 2:**
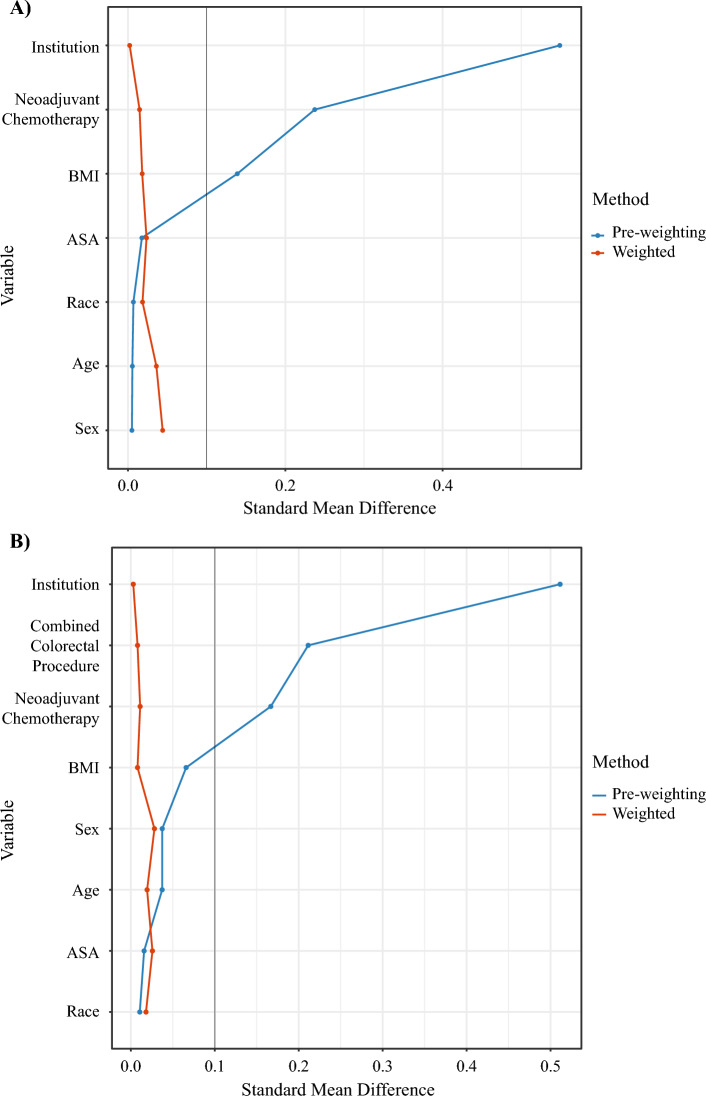
Comparison of standard mean differences pre- and post-matching weights for **A** patients who underwent HAIP placement alone (*n* = 445 pre-weighting) and **B** the entire cohort (*n* = 819 pre-weighting). *HAIP* Hepatic artery infusion pump, *BMI* Body mass index, *ASA* American Society of Anesthesiologists

After PSMW was performed, the robotic and open cohorts were well balanced, with a standard mean difference of < 0.1 for all matched variables in the cohort that underwent HAIP placement alone without a colorectal or other procedure (Fig. 2a) and in the entire cohort (Fig. 2b). Both unadjusted and adjusted values for the primary outcomes are reported in Table 2.

The comparison of EBL, operative time, and LOS was restricted to patients who underwent HAIP placement alone without a combined colorectal or other procedure to eliminate confounding (Fig. 2a and Table 2). The robotic approach was associated with a 68-min (95% confidence interval [CI] +55 to +81, *p* < 0.001) longer operative time; after PSMW, the median duration of operative time was 225 min (IQR 197–255) for the robotic approach versus 155 min (IQR 131–182) for the open approach. LOS was approximately 2 days shorter in the robotic cohort (− 1.8 days, 95% CI − 2.3 to − 1.3, *p* < 0.001). Following PSMW, the median LOS was 3 days (IQR 2–4) after robotic HAIP placement versus 5 days (IQR 4–6) after open placement. There was no difference in EBL (*p* = 0.2).

**Fig. 3:**
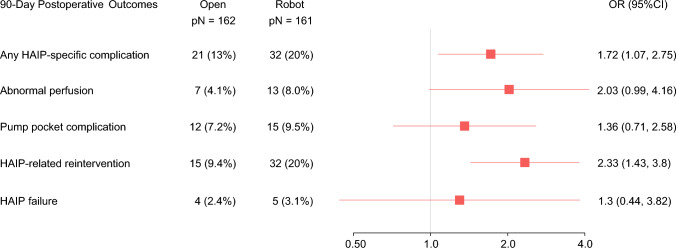
Forest plots of 90-day postoperative outcomes from propensity score matching weight analysis. *OR* Odds ratio, *CI* Confidence interval, *HAIP* Hepatic artery infusion pump

Analysis of HAIP-specific complications and 90-day postoperative outcomes included the entire study cohort, and a combined colorectal procedure variable was included in PSMW (Fig. 2b). HAIP-specific complications were more common following robotic HAIP placement (odds ratio [OR] 1.72, 95% CI 1.07–2.75, *p* = 0.025), with significantly more patients requiring an additional HAIP-related procedure within 90 days (OR 2.33, 95% CI 1.43–3.80, *p* < 0.001) (Table 2, Fig. 3). We observed twice the incidence of abnormal perfusion requiring re-intervention in the robotic cohort compared with the open cohort (PSMW-adjusted incidence of 8.0% vs. 4.1%, *p* = 0.054). Pump pocket complications were observed in 9.5% of patients who underwent robotic HAIP implantation and 7.2% of patients who had open HAIP implantation (*p* = 0.4). The time to initiation of HAIP chemotherapy and odds of 90-day HAIP failure due to technical complications were not significantly different (*p* = 0.4 and *p* = 0.6, respectively) when comparing the robotic cohort with the open cohort (Table 2).

## Discussion

In this study, we present the largest series of HAIP placements reported to date, offering a contemporary update on the risk of HAIP-specific complications in the postoperative period, and a comparison of outcomes between robotic and open placement. After adjusting for demographic and clinical factors, receipt of neoadjuvant chemotherapy, institution, and the higher prevalence of combined colorectal procedures in open cases, we found robotic HAIP placement is associated with longer operative time but shorter LOS compared with the traditional open approach. While we observed significantly more HAIP-specific complications and re-intervention within 90 days of robotic HAIP placement, the odds of pump failure and time to initiation of HAIP chemotherapy were not significantly different. Overall, 15% of patients experienced an HAIP-specific complication, 12% had an HAIP-related re-intervention, and 2.7% experienced HAIP failure due to an early complication within 90 days of placement. While no deaths with documentation of a proximal cause were attributable to an HAIP-specific complication, 4% of patients died within 90 days postoperatively, most often related to progression of disease.

Our finding of decreased LOS in robotic placement is consistent with the most recently reported series from The Netherlands describing early outcomes of HAIP placement.^20^ Ten Haaft et al. compared 22 robotic and 28 open HAIP placements for intrahepatic cholangiocarcinoma, finding robotic HAIP placement had a shorter median LOS of 3 days (IQR 2–4) compared with 6 days (IQR 4–9) in open procedures, similar to our PSMW-adjusted median LOS of 3 and 5 days, respectively.

Ten Haaft et al. also found no difference in overall grade 3 or higher complications (18% robotic vs. 21% open, *p* = 1.0) and observed only one patient each in the robotic (5%) and open (4%) cohorts with any catheter, arterial, or perfusion-related complication; pump pocket complications were not reported. The present series includes cases that were performed during the development phase of robotic pump implantation, a technique that was refined, optimized, and since adopted by other centers, including those in the Netherlands, which may account for their lower observed incidence of catheter complications. In the landmark series of 544 open HAIP placements at MSK reported by Allen et al. in 2005, the lifetime risk of HAIP-specific complications was 22%, with 8% of patients experiencing an early complication within 30 days and 5% experiencing pump failure within 6 months.^21^ The robotic series by Dhir et al. at the University of Pittsburgh reported a 30-day complication rate of 8% and lifetime rate of 21%.^19^ As we used a longer time period to measure early postoperative complications (90 days), our findings of 15% complication and 3% failure rates are in the range of these prior estimates.

While we grouped all HAIP-related complications into a single endpoint, it is important to note specific complications drove higher postoperative morbidity—pump pocket issues were the most common category of complications, followed by abnormal perfusion. These two categories of complications were also the major drivers of early postoperative re-intervention. Arterial dissection and bleeding from the cannulation site were rare in the early postoperative period. These findings are similar to the early (1986–2001) MSK experience, with the notable exception that no cases of early arterial thrombosis were observed in this modern cohort.^21^ We observed double the incidence of abnormal perfusion in the robotic cohort compared with the open cohort, albeit not significant on PSMW analysis; pump pocket complications were also higher but not statistically different. The higher rate of re-intervention in the robotic cohort was driven primarily by IR procedures necessary to salvage HAIP with abnormal perfusion and operative re-exploration for pump pocket issues. While not conclusive, our data suggest surgeons adopting the robotic approach to HAIP placement remain attentive to a potentially increased risk of abnormal perfusion and pump pocket complications with robotic placement. As the technical dissection and intraoperative evaluation of perfusion are not different between robotic and open placement, these findings may reflect the unmeasured impact of the learning curve. The steps involved in creation of the pump pocket are not performed robotically and are ostensibly identical. The observed differences in pump pocket complications were primarily driven by a higher number of hematomas and seromas in the robotic group. This raises the question of whether differences in technique (e.g., size of the pump pocket, evaluation for hemostasis in the setting of positive intra-abdominal pressure) could exist.

The median operative time for robotic placement (225 min) in our cohort is similar to the two other published series (222–233 min), which also describe a 4–5% rate of conversion from robotic to open.^19,20^ This conversion rate compares favorably to the previously reported laparoscopic experience at MSK, in which 67% of attempted laparoscopic cases were converted to open.^17^ Furthermore, while no patients died of an HAIP-specific complication, and 30-day mortality was 0.5%, the 90-day mortality rate of 4% suggests room to improve patient selection, as most deaths were due to progression of disease.

Our study has several limitations. First, the choice of operative approach was surgeon-dependent and not randomized. While we used PSMW to try to balance differences between groups, our analysis is still retrospective in nature. There was a higher rate of combined colorectal procedures in the open cohort (45% vs. 34%, *p* = 0.011). To account for this potential confounder, combined procedures were excluded from comparisons of operative time, EBL, and LOS, and the cohorts were well balanced after application of PSMW (Fig. 2b) to adjust for combined colorectal procedure for the comparison of HAIP-specific complications, re-intervention, time to initiation of HAIP chemotherapy, and HAIP failure. Second, our study included cases performed during the learning period when the robotic approach was first developed at MSK, adopted by new surgeons at MSK, and implemented as part of a new HAIP program at Duke. A previous analysis of the learning curve in open HAIP placement found a reduction in lifetime complications from 31% to 19% after individual surgeons had performed at least 25 open HAIP placements.^21^ While inclusion of cases on the initial trajectory of the learning curve may have biased towards a higher rate of complications, it ensures our findings are a relevant benchmark for surgeons starting new HAIP therapy programs.

Our study is also limited by the exclusion of several important outcomes we were unable to assess that will impact the balance when weighing harms versus potential benefits of the robotic approach. The first of these involves differences in cost—potential savings from a nearly 2-day reduction in LOS could outweigh the added cost of robotic instruments and longer operative time, and may be offset by the cost of managing additional complications. Second, while we observed a reduction in LOS, patient-reported outcomes would better measure the benefit of expediting functional recovery to patients, including the impact on quality of life. Third, the only oncologic outcome measured in our study was return to intended therapy (time to initiation of HAIP chemotherapy); we did not measure duration of HAIP chemotherapy, dose reductions, or long-term outcomes, including biliary sclerosis. While the faster functional recovery is reflected in decreased LOS, this did not translate to earlier initiation of HAIP chemotherapy. This may be due to the need for additional procedures (e.g., to correct abnormal perfusion) in the robotic cohort or simply that medical oncologists have maintained the same time frame for initiation of therapy regardless of operative approach based on established practice. Finally, using a minimally invasive approach for HAIP placement can have a significant impact on the ease of subsequent operations. While impossible to quantify, subsequent liver resections can more easily be performed after robotic HAIP placement compared with the open approach, in the authors’ experience. Minimizing the physiologic impact and scarring with each operation is particularly important for patients with colorectal liver metastasis, who may require multiple operations to manage their primary tumor and liver metastasis over a long course of disease.

## Conclusions

The rate of early postoperative HAIP-specific complications and re-intervention may be higher following robotic placement; however, the increase in complications did not impact the initiation of HAIP chemotherapy in our study. Furthermore, robotic placement was associated with significantly shorter LOS, highlighting the potential benefit of expedited functional recovery. As the use of robotic procedures continues to grow, additional evaluations that integrate cost, patient-reported outcomes, and the impact of the learning curve are needed.
